# Effectiveness of enhanced recovery after surgery protocols in elderly patients undergoing major abdominal surgery: a systematic review and meta-analysis

**DOI:** 10.3389/fsurg.2026.1823500

**Published:** 2026-05-21

**Authors:** Kexuan Zhu, Qingkun Zhou, Xuhui Xu, Zhengdong Wu, Kunfeng Ban, Gang Xu

**Affiliations:** 1 School of Medicine and Nursing, Nanjing University of Chinese Medicine Hanlin College, Taizhou, Jiangsu, China; 2 Department of Geriatrics, Nanjing Tongren Hospital, School of Medicine, Southeast University, Nanjing, Jiangsu, China; 3 Department of General Surgery, Nanjing Tongren Hospital, School of Medicine, Southeast University, Nanjing, Jiangsu, China

**Keywords:** abdominal surgery, elderly, enhanced recovery after surgery, ERAS, geriatric, meta-analysis, systematic review

## Abstract

**Background:**

Enhanced Recovery After Surgery (ERAS) protocols have been widely adopted in perioperative care, but their effectiveness and safety in elderly patients undergoing major abdominal surgery remain subjects of ongoing investigation. To systematically evaluate the effectiveness of ERAS protocols compared with conventional care in elderly patients (≥65 years) undergoing major abdominal surgery.

**Methods:**

A systematic search was conducted in PubMed, Cochrane Library, Embase, and Web of Science from inception to December 2024. Randomized controlled trials (RCTs) and prospective cohort studies comparing ERAS with conventional care in elderly patients undergoing major abdominal surgery were included. The primary outcome was length of hospital stay (LOS). Secondary outcomes included total complication rate, 30-day mortality, and 30-day readmission rate. ERAS components, protocol compliance, baseline characteristics, and complication definitions were extracted when available. Meta-analyses were performed using random-effects models with subgroup and sensitivity analyses.

**Results:**

Fifteen studies (6 RCTs and 9 prospective cohort studies) involving 2,397 patients were included. Most studies evaluated colorectal surgery, while two involved hepatectomy and two involved gastrectomy. ERAS pathways consistently included early feeding, early mobilization, and reduced routine tube use, but reporting of protocol details and compliance was variable; overall compliance was reported in 6 of 15 studies (range 42%–89.6%). ERAS significantly reduced LOS compared with conventional care (MD = −3.31 days, 95% CI: −3.74 to −2.88, *P* < 0.0001). ERAS was associated with a significantly lower complication rate (RR = 0.63, 95% CI: 0.56–0.71, *P* < 0.0001) and a borderline reduction in 30-day mortality (RR = 0.53, 95% CI: 0.28–1.00, *P* = 0.049). No significant difference was observed in 30-day readmission rate (RR = 0.78, 95% CI: 0.56–1.08, *P* = 0.135). Sensitivity analysis excluding the one non-elderly hepatectomy trial yielded materially similar results.

**Conclusions:**

ERAS protocols appear effective and generally safe in older patients undergoing major abdominal surgery, reducing hospital stay and postoperative complications without increasing readmission rates. However, interpretation should account for variability in ERAS implementation, incomplete compliance reporting, and the combination of randomized and prospective cohort evidence.

**Systematic Review Registration:**

https://www.crd.york.ac.uk/PROSPERO/view/CRD420261381194, PROSPERO CRD420261381194.

## Introduction

The global population is aging rapidly, with projections indicating that individuals aged 65 years and older will constitute over 20% of the world population by 2050 (1). This demographic shift has profound implications for surgical practice, as elderly patients increasingly require major abdominal surgical interventions for conditions including colorectal cancer, hepatobiliary diseases, and gastric malignancies (2, 3). However, advanced age is associated with increased perioperative risks, including higher rates of postoperative complications, prolonged hospital stays, and greater healthcare resource utilization (4).

Enhanced Recovery After Surgery (ERAS) protocols, first introduced by Kehlet in the 1990s, represent a multimodal, evidence-based approach to perioperative care designed to attenuate the surgical stress response and accelerate functional recovery (5, 6). These protocols typically incorporate preoperative optimization, intraoperative interventions, and postoperative measures including early mobilization, early oral feeding, and multimodal analgesia (7). ERAS has demonstrated significant benefits in the general surgical population, including reduced length of hospital stay, decreased complication rates, and lower healthcare costs (8, 9).

Despite these benefits, concerns have been raised regarding the applicability of ERAS protocols to elderly patients, who often present with multiple comorbidities, reduced physiological reserve, and increased frailty (10, 11). Some clinicians have questioned whether aggressive early mobilization and feeding protocols might be too demanding for this vulnerable population, potentially leading to adverse events or increased readmission rates (12). Conversely, proponents argue that elderly patients may derive even greater benefits from ERAS due to the potential reduction in complications such as postoperative delirium, pneumonia, and venous thromboembolism (13).

Recent guidelines from the European Association for Endoscopic Surgery (EAES) and the Society of American Gastrointestinal and Endoscopic Surgeons (SAGES) have emphasized the importance of optimizing perioperative care in older adults (14). However, the evidence base specifically addressing ERAS implementation in elderly patients undergoing major abdominal surgery requires systematic evaluation. Previous meta-analyses have either focused on the general population or included limited numbers of elderly-specific studies (15, 16).

The objective of this systematic review and meta-analysis was to comprehensively evaluate the effectiveness and safety of ERAS protocols compared with conventional perioperative care in elderly patients (≥65 years) undergoing major abdominal surgery, with specific focus on length of hospital stay, postoperative complications, 30-day mortality, and 30-day readmission rates.

## Materials and methods

### Protocol and registration

This systematic review and meta-analysis was conducted following the Preferred Reporting Items for Systematic Reviews and Meta-Analyses (PRISMA) 2020 guidelines (17). The protocol was developed *a priori* following the Cochrane Handbook for Systematic Reviews of Interventions (18). This review was prospectively registered in PROSPERO (registration number: CRD420261381194).

### Eligibility criteria

Studies were included if they met the following criteria: (1) randomized controlled trials (RCTs) or prospective cohort studies; (2) included elderly patients aged ≥65 years or with a mean age ≥65 years undergoing major abdominal surgery; (3) compared ERAS or fast-track surgery protocols with conventional perioperative care; (4) reported at least one of the following outcomes: length of hospital stay, total complication rate, 30-day mortality, or 30-day readmission rate; and (5) published in English. Studies were excluded if they were retrospective in design, case reports, case series, review articles, conference abstracts without full-text availability, or included pediatric or young adult populations exclusively.

### Information sources and search strategy

A comprehensive literature search was conducted in PubMed, Cochrane Library, Embase, and Web of Science from database inception through December 2024. The search strategy combined Medical Subject Headings (MeSH) terms and free-text keywords related to: (1) enhanced recovery after surgery, ERAS, fast-track surgery, or enhanced recovery program; (2) elderly, older, aged, geriatric, or senior; and (3) abdominal surgery, colorectal surgery, gastrointestinal surgery, hepatectomy, pancreatic surgery, or gastric surgery. Reference lists of included studies and relevant reviews were manually searched to identify additional eligible studies.

### Study selection and data extraction

Two reviewers independently screened titles and abstracts for eligibility, followed by full-text review of potentially relevant articles. Discrepancies were resolved through discussion and consensus. Data extraction was performed using a standardized form including: study characteristics (author, year, country, study design), participant characteristics (sample size, age, sex distribution, and baseline characteristics such as ASA grade or comorbidity indices when reported), intervention details (ERAS protocol components and protocol compliance), surgical details (type of abdominal procedure), and outcomes (length of stay, complication rates, mortality, readmission rates). Complication definitions and the specific complications reported by each study were also collected whenever available. For continuous outcomes reported as medians with interquartile ranges, means and standard deviations were estimated using established methods.

### Quality assessment

Risk of bias in RCTs was assessed using the Cochrane Risk of Bias Tool 2.0 (19). Quality of non-randomized studies was evaluated using the Newcastle-Ottawa Scale (NOS) (20). The overall certainty of evidence was assessed using the Grading of Recommendations Assessment, Development and Evaluation (GRADE) framework (21).

### Statistical analysis

Meta-analyses were performed using random-effects models with the inverse variance method. For continuous outcomes (length of hospital stay), mean differences (MD) with 95% confidence intervals (CI) were calculated. For dichotomous outcomes (complications, mortality, readmission), risk ratios (RR) with 95% CI were computed. Statistical heterogeneity was assessed using the Cochran Q test and quantified using the I² statistic, with I² values of 25%, 50%, and 75% representing low, moderate, and high heterogeneity, respectively (22). Subgroup analyses were performed according to study design (RCT vs. prospective cohort). Sensitivity analyses were conducted using the leave-one-out method. Publication bias was assessed using funnel plots and Egger's regression test for outcomes with ≥10 studies (23). Statistical analyses were performed using R version 4.3.3 with the meta package. A two-sided *P* value < 0.05 was considered statistically significant.

## Results

### Study selection

**Figure 1:**
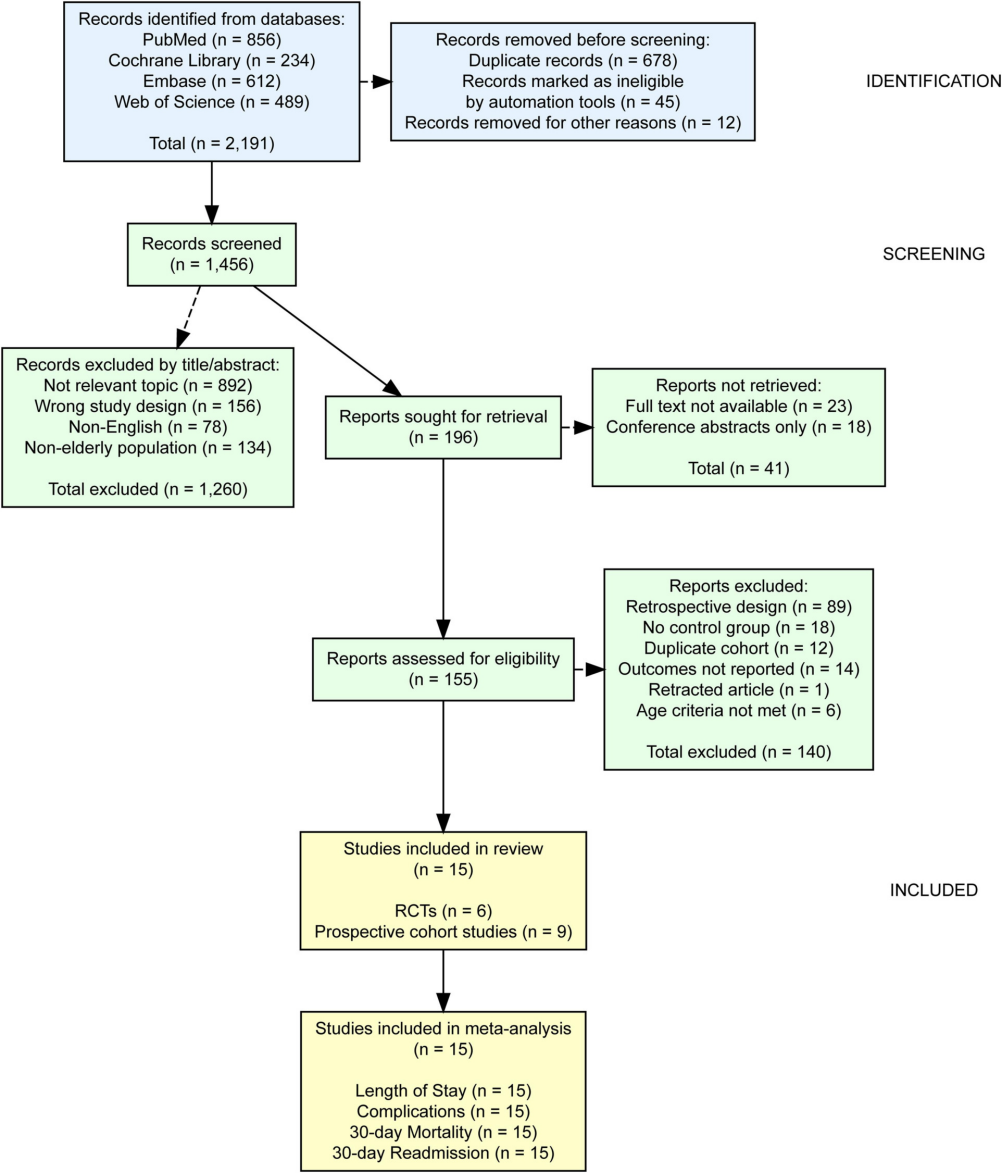
PRISMA 2020 flow diagram showing the study selection process.

The systematic search identified 2,191 records from four databases. After removing 678 duplicates and 57 records marked as ineligible by automation tools or other reasons, 1,456 records were screened by title and abstract. Of these, 1,260 were excluded, leaving 196 reports for full-text retrieval. Forty-one reports could not be retrieved, and 155 were assessed for eligibility. After excluding 140 studies (89 retrospective designs, 18 lacking control groups, 12 duplicate cohorts, 14 not reporting outcomes of interest, 6 not meeting age criteria, and 1 retracted article), 15 studies were included in the final analysis (Figure 1).

### Study characteristics

The 15 included studies comprised 6 RCTs (24–29) and 9 prospective cohort studies (30–38) published between 2007 and 2021, enrolling a total of 2,397 patients (1,200 in ERAS groups and 1,197 in control groups). Studies were conducted across multiple countries including China (*n* = 5), Switzerland (*n* = 1), Italy (*n* = 1), Norway (*n* = 1), Germany (*n* = 1), Spain (*n* = 3), UK (*n* = 1), and Poland (*n* = 2). The surgical case-mix was predominantly colorectal surgery (11 studies), with 2 hepatectomy studies and 2 gastrectomy studies. Age thresholds ranged from ≥65 to ≥80 years, although one hepatectomy trial enrolled younger adults and one cohort included a mixed adult population; both were retained because the perioperative comparison remained relevant, and sensitivity analyses were performed to assess their influence. Detailed study characteristics and baseline variables are presented in Table 1 and Supplementary Table S1.

**Table 1:** Characteristics of included studies.

Study	Year	Country	Design	ERAS (*n*)	Control (*n*)	Surgery type
Ostermann et al. (24)	2019	Switzerland	RCT	77	73	Colorectal
Wang et al. (25)	2012	China	RCT	40	38	Lap colorectal
Jia et al. (26)	2014	China	RCT	117	116	Open colorectal
Cao et al. (27)	2021	China	RCT	85	86	Lap gastrectomy
Qi et al. (28)	2018	China	RCT	80	80	Hepatectomy
Liu et al. (29)	2016	China	RCT	42	42	Gastrectomy
Lirosi et al. (30)	2019	Italy	Prospective cohort	61	53	Colorectal
Forsmo et al. (31)	2017	Norway	Prospective cohort	75	78	Colorectal
Scharfenberg et al. (32)	2007	Germany	Prospective cohort	74	68	Open colonic
Jiang et al. (33)	2020	China	Prospective cohort	70	107	Lap hepatectomy
Tejedor et al. (34)	2018	Spain	Prospective cohort	156	156	Colorectal
Gonzalez-Ayora et al. (35)	2016	Spain	Prospective cohort	188	175	Colorectal
Walter et al. (38)	2014	UK	Prospective cohort	52	48	Colorectal
Pedziwiatr et al. (36)	2016	Poland	Prospective cohort	45	42	Lap colorectal
Kisialeuski et al. (37)	2015	Poland	Prospective cohort	38	35	Colorectal

RCT, randomized controlled trial; Lap, laparoscopic.

Across studies, ERAS pathways most commonly included preoperative counseling, avoidance of prolonged fasting or bowel preparation, goal-directed fluid management, multimodal analgesia, early oral intake, early mobilization, and early urinary catheter removal. However, the exact combination of elements differed across trials, reflecting local pathway design and publication era. A detailed cross-study summary of ERAS elements is provided in Supplementary Table S2.

Protocol adherence was incompletely reported. Overall compliance rates were available in 6 of 15 studies and ranged from 42% to 89.6%, while 9 studies did not report a global compliance rate. Where available, adherence appeared generally acceptable for core items such as early intake and mobilization, but the absence of uniform compliance reporting limits interpretation of implementation fidelity (Supplementary Table S3).

### Primary outcome: length of hospital stay

**Figure 2:**
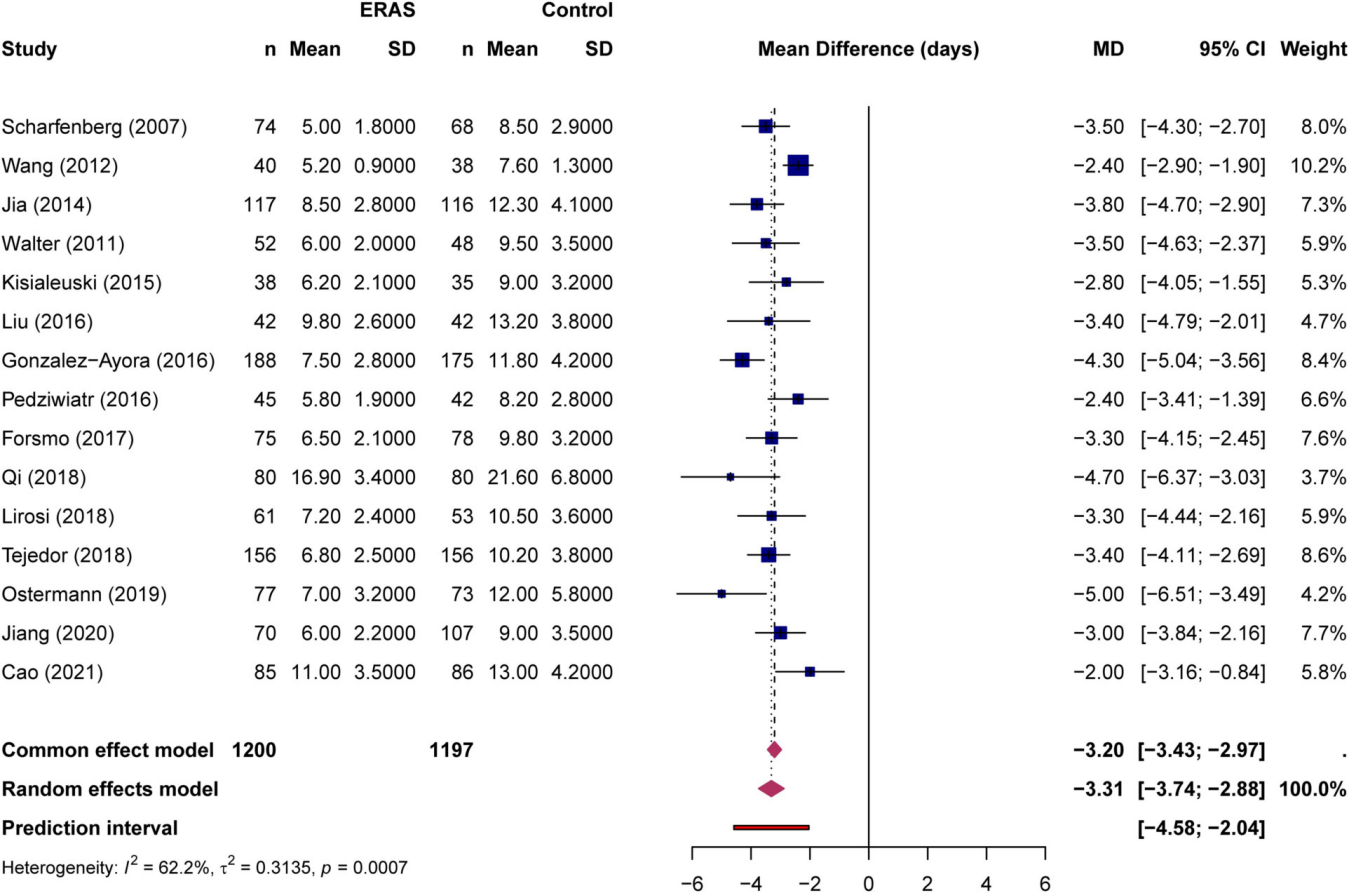
Forest plot for length of hospital stay comparing ERAS with conventional care.

All 15 studies reported length of hospital stay data. Meta-analysis demonstrated that ERAS protocols significantly reduced LOS compared with conventional care (MD = −3.31 days, 95% CI: −3.74 to −2.88, *P* < 0.0001; Figure 2). Moderate heterogeneity was observed (I² = 62.2%, *P* = 0.0007). From a clinical perspective, an average reduction of approximately 3.3 hospital days is likely meaningful for older patients because it may reduce immobilization-related complications, resource use, and exposure to nosocomial events.

**Figure 3:**
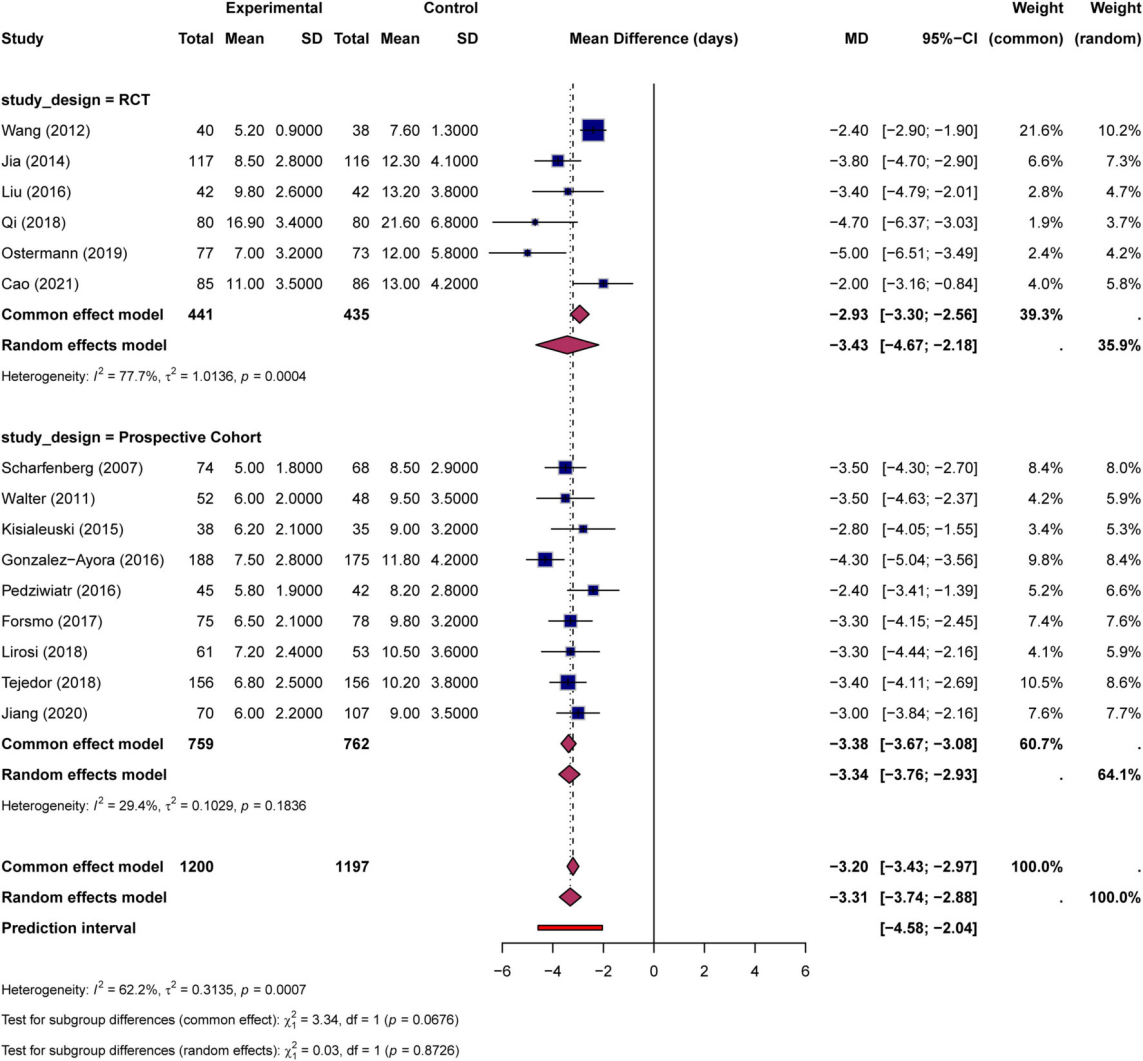
Forest plot for length of hospital stay by study design subgroup (RCT vs. Prospective Cohort).

Subgroup analysis by study design revealed consistent effects across study types (Figure 3). In the RCT subgroup (6 studies, 441 ERAS patients and 435 controls), the pooled MD was −3.43 days (95% CI: −4.67 to −2.18), with high heterogeneity (I² = 77.7%, *P* = 0.0004). In the prospective cohort subgroup (9 studies, 759 ERAS patients and 762 controls), the pooled MD was −3.34 days (95% CI: −3.76 to −2.93), with low heterogeneity (I² = 29.4%, *P* = 0.184). The test for subgroup differences was not statistically significant under the random-effects model (*χ*² = 0.03, *P* = 0.873), indicating that the beneficial effect of ERAS on LOS was broadly consistent regardless of study design. Plausible contributors to LOS heterogeneity include differences in surgical complexity (for example, colorectal resection vs. hepatectomy or gastrectomy), variation in ERAS elements and adherence, conversion of medians to means in several studies, and differences in local discharge criteria. Sensitivity analysis using the leave-one-out method demonstrated robust results, with pooled estimates ranging from −3.21 to −3.41 days after sequential exclusion of individual studies (Table 2). Excluding the one non-elderly hepatectomy trial also produced a nearly identical pooled estimate (MD = −3.26 days, 95% CI: −3.66 to −2.86; Supplementary Table S5).

**Table 2:** Summary of meta-analysis results.

Outcome	Studies	Patients	Effect estimate	95% CI	*P* value	I² (%)
Length of stay (days)	15	2,397	MD = −3.31	−3.74 to −2.88	<0.0001	62.2
RCT subgroup	6	876	MD = −3.43	−4.67 to −2.18	<0.0001	77.7
Cohort subgroup	9	1,521	MD = −3.34	−3.76 to −2.93	<0.0001	29.4
Total complications	15	2,397	RR = 0.63	0.56–0.71	<0.0001	0
RCT subgroup	6	876	RR = 0.52	0.42–0.65	<0.0001	0
Cohort subgroup	9	1,521	RR = 0.67	0.58–0.78	<0.0001	0
30-Day mortality	15	2,397	RR = 0.53	0.28–1.00	0.049	0
30-Day readmission	15	2,397	RR = 0.78	0.56–1.08	0.135	0

MD, mean difference; RR, risk ratio; CI, confidence interval; RCT, randomized controlled trial.

### Secondary outcomes

#### Total complication rate

**Figure 4:**
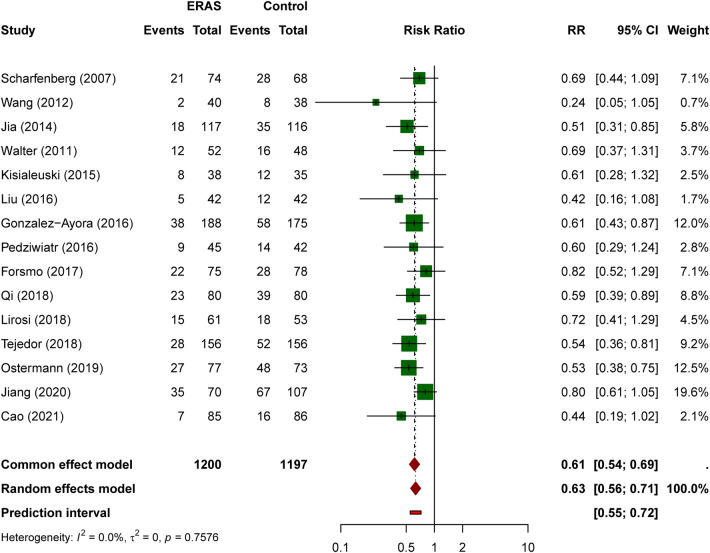
Forest plot for total complication rate comparing ERAS with conventional care.

Fifteen studies reported total complication rates, with 270 events among 1,200 ERAS patients (22.5%) vs. 451 events among 1,197 control patients (37.7%). ERAS protocols were associated with a significantly reduced complication rate (RR = 0.63, 95% CI: 0.56–0.71, *P* < 0.0001; Figure 4). In most studies, complications referred to overall postoperative morbidity rather than only reoperation or major surgical events. Reported events included both surgical complications (such as anastomotic leak, wound infection, obstruction, ileus, or abscess) and medical complications (such as pneumonia, urinary tract infection, delirium, venous thromboembolism, and cardiac events). Eight studies used the Clavien-Dindo classification system, whereas the remainder reported overall morbidity using study-specific definitions (Supplementary Table S4). No significant heterogeneity was observed (I² = 0%, *P* = 0.758), indicating consistent effects across studies.

**Figure 5:**
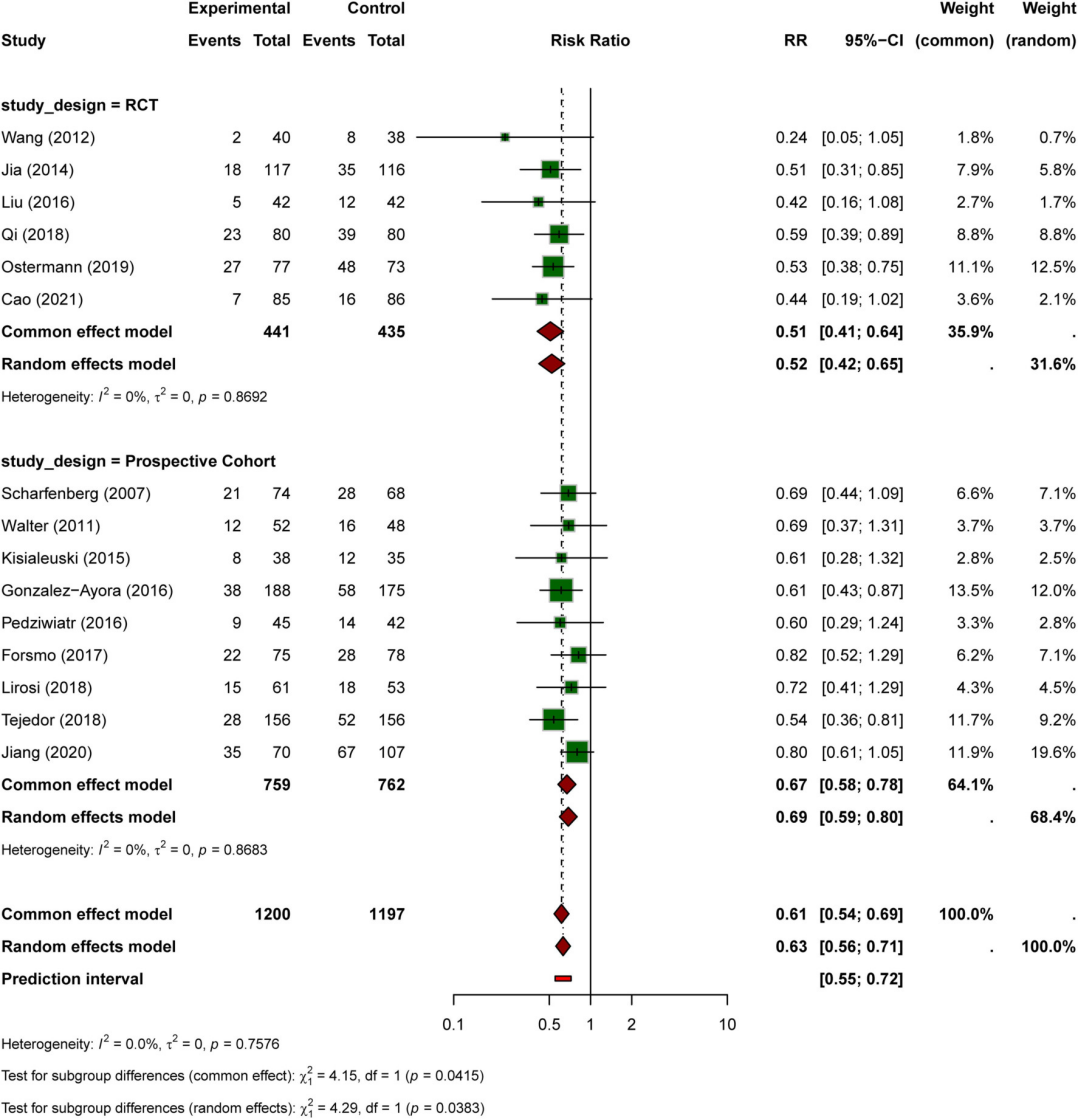
Forest plot for total complication rate by study design subgroup (RCT vs. Prospective Cohort).

Subgroup analysis by study design showed that the complication-reducing effect of ERAS was more pronounced in RCTs than in prospective cohort studies (Figure 5). In the RCT subgroup (6 studies), the pooled RR was 0.52 (95% CI: 0.42–0.65, I² = 0%, *P* = 0.869). In the prospective cohort subgroup (9 studies), the pooled RR was 0.67 (95% CI: 0.58–0.78, I² = 0%, *P* = 0.868). Notably, the test for subgroup differences reached statistical significance (*χ*² = 4.29, *P* = 0.038 under the random-effects model), suggesting that the magnitude of effect may differ between RCTs and observational studies, although both consistently favored ERAS over conventional care.

#### 30-day mortality

**Figure 6:**
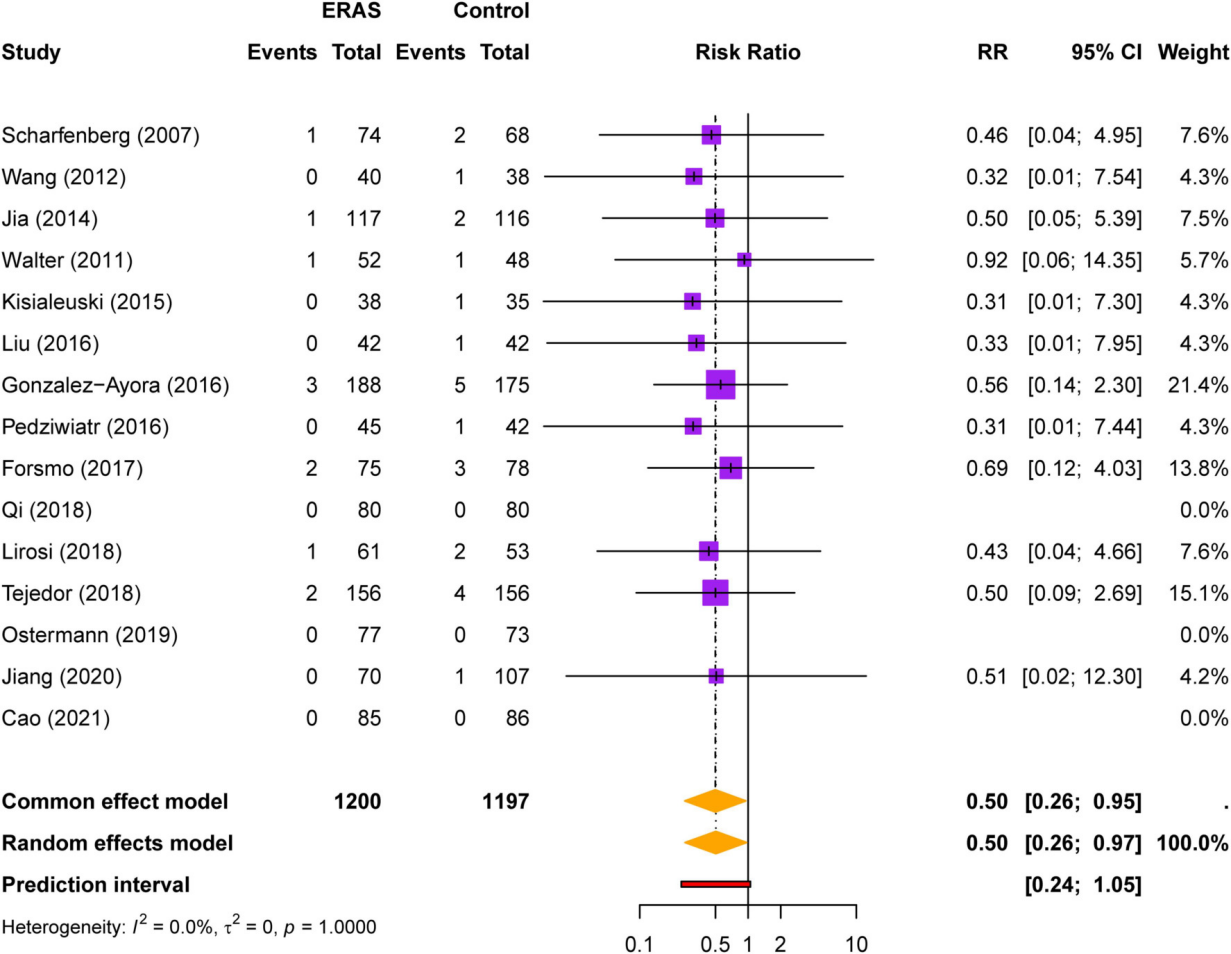
Forest plot for 30-day mortality comparing ERAS with conventional care.

Fifteen studies reported 30-day mortality data. Overall mortality was low, with 11 deaths among 1,200 ERAS patients (0.92%) compared with 24 deaths among 1,197 control patients (2.01%). ERAS was associated with a borderline significant reduction in 30-day mortality (RR = 0.53, 95% CI: 0.28–1.00, *P* = 0.049; Figure 6). No heterogeneity was detected (I² = 0%). Given the low event rate and a confidence interval reaching 1.00, this result should be interpreted cautiously and considered hypothesis-generating rather than definitive proof of a mortality benefit.

#### 30-day readmission rate

**Figure 7:**
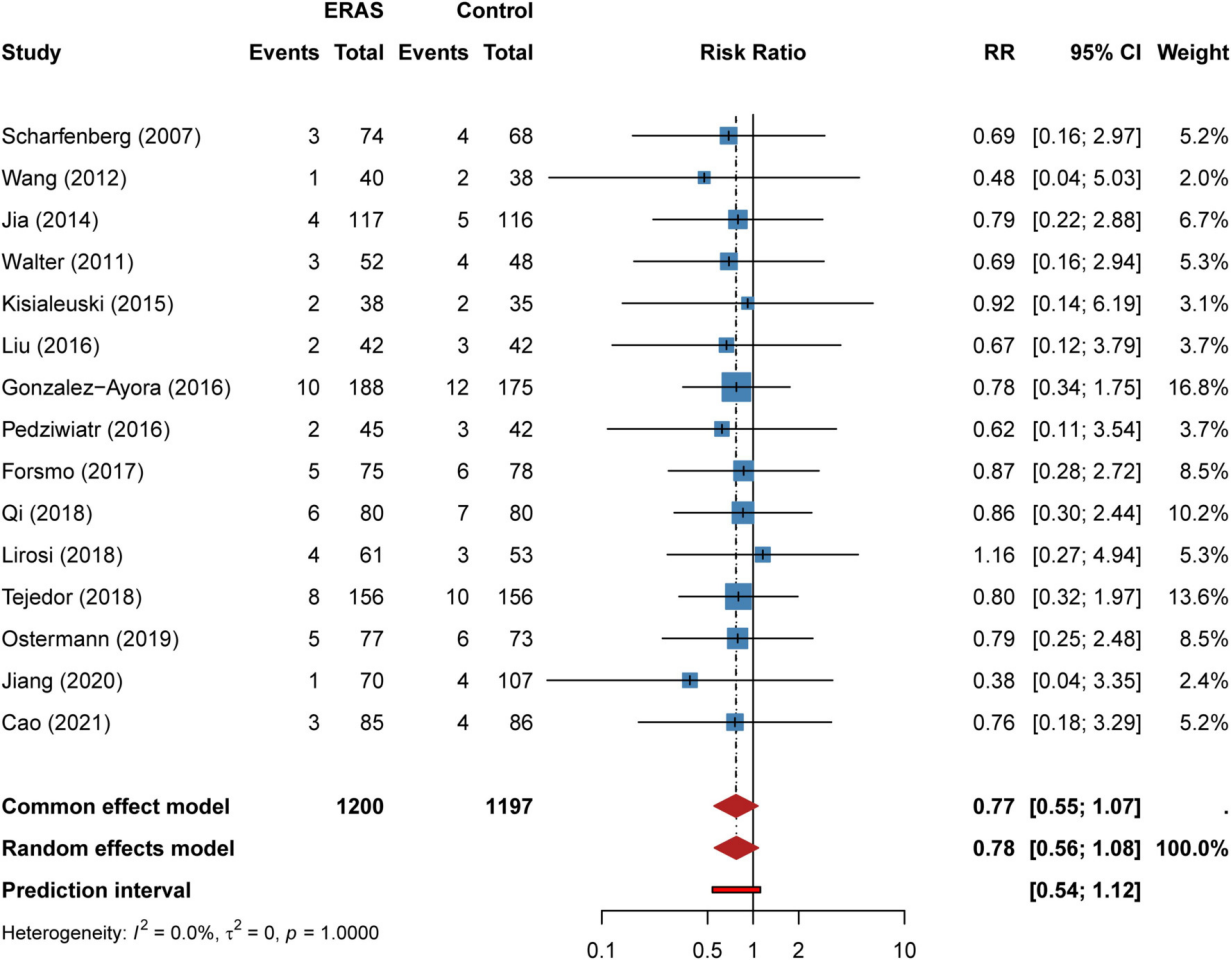
Forest plot for 30-day readmission rate comparing ERAS with conventional care.

Fifteen studies reported 30-day readmission rates. Readmission occurred in 59 of 1,200 ERAS patients (4.92%) and 75 of 1,197 control patients (6.27%). No significant difference was observed between groups (RR = 0.78, 95% CI: 0.56–1.08, *P* = 0.135; Figure 7). Heterogeneity was absent (I² = 0%), confirming that ERAS does not increase readmission risk in elderly patients.

### Quality assessment and publication bias

**Figure 8:**
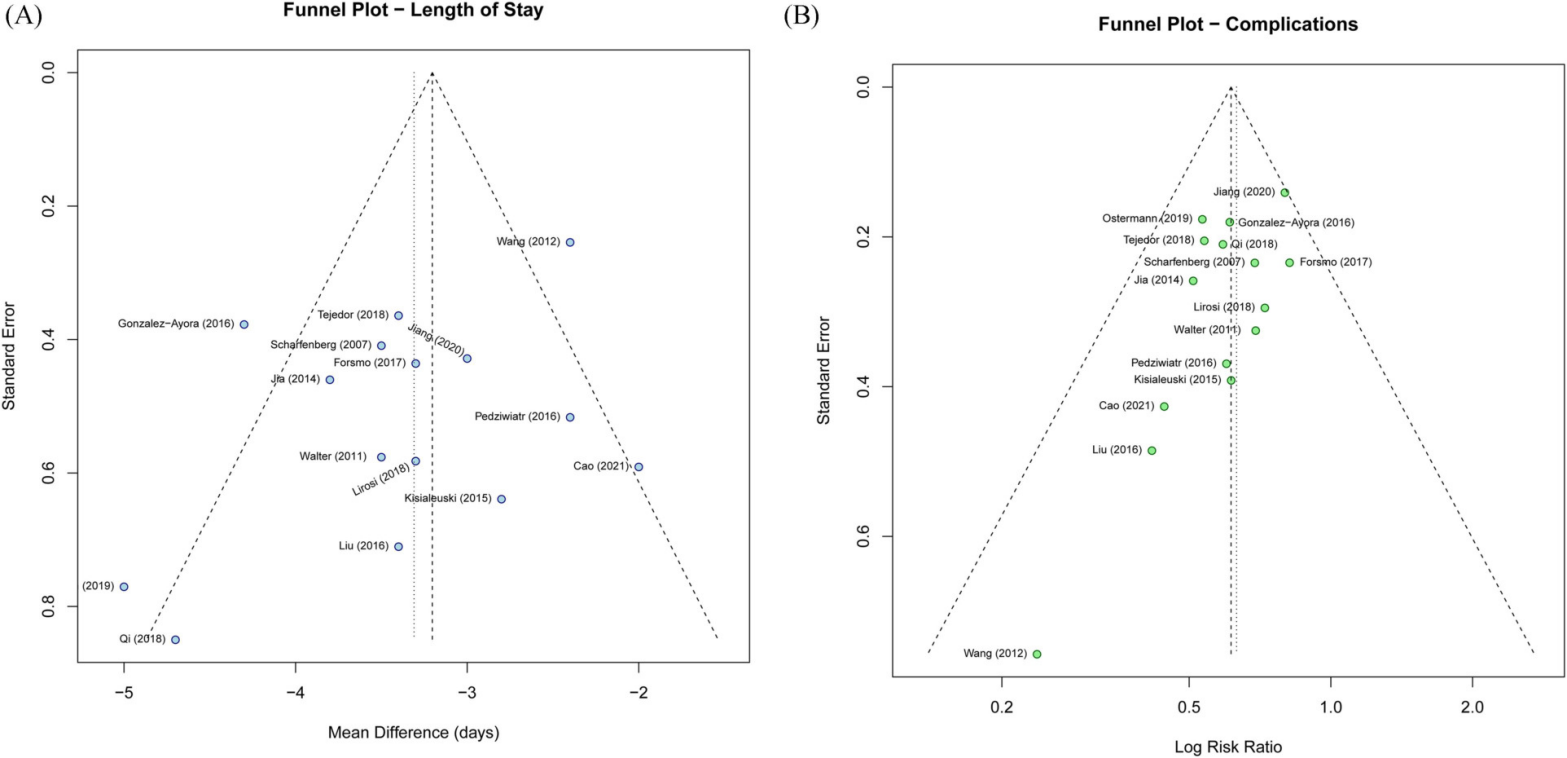
Funnel plots for publication bias assessment. **(A)** Length of hospital stay. **(B)** Total complication rate.

Risk of bias assessment revealed that all RCTs had some concerns regarding blinding of participants and personnel, which is inherent to the nature of perioperative care interventions. Prospective cohort studies generally scored 6–7 points on the Newcastle-Ottawa Scale, indicating moderate to high quality. The funnel plot for length of hospital stay showed some asymmetry (Figure 8A), and Egger's test indicated potential publication bias (*P* = 0.0015). However, the trim-and-fill analysis suggested that even accounting for potentially missing studies, the overall conclusion remained unchanged. The funnel plot for complications appeared symmetric (Figure 8B), with Egger's test showing no significant asymmetry (*P* = 0.312). The GRADE assessment indicated moderate certainty of evidence for length of stay and complications, and low certainty for mortality and readmission outcomes due to imprecision (Table 3). For LOS, certainty was downgraded for inconsistency because heterogeneity was moderate overall and substantial within the RCT subgroup, likely reflecting differences in surgical case-mix, ERAS implementation, and discharge practice rather than a reversal of treatment direction.

**Table 3:** GRADE evidence quality assessment.

Outcome	Risk of bias	Inconsistency	Indirectness	Imprecision	Pub. bias	Certainty
Length of stay	Moderate	Serious	Not serious	Not serious	Suspected	Moderate
Complications	Moderate	Not serious	Not serious	Not serious	Unlikely	Moderate
30-day mortality	Moderate	Not serious	Not serious	Serious	Unlikely	Low
30-day readmission	Moderate	Not serious	Not serious	Serious	Unlikely	Low

GRADE, grading of recommendations assessment, development and evaluation; Pub., publication.

## Discussion

This systematic review and meta-analysis provides comprehensive evidence supporting the effectiveness and safety of ERAS protocols in elderly patients undergoing major abdominal surgery. Our findings demonstrate that ERAS implementation results in clinically meaningful reductions in hospital length of stay (approximately 3.3 days) and a 37% relative reduction in postoperative complication rates, with no evidence of increased readmission. The observed reduction in mortality should be interpreted with caution because the absolute number of events was small and the confidence interval approached the null.

The magnitude of LOS reduction observed in our analysis (MD = −3.31 days) is consistent with previous meta-analyses examining ERAS in the general surgical population (39, 40). Importantly, subgroup analyses demonstrated that this benefit was consistent across both RCTs (MD = −3.43 days) and prospective cohort studies (MD = −3.34 days), with no significant subgroup difference (*P* = 0.873). The moderate-to-high heterogeneity for LOS is clinically plausible, because pooling studies across colorectal, hepatobiliary, and gastric procedures necessarily combines operations with different expected recovery trajectories. In addition, ERAS pathways were not identical across studies, and compliance reporting was often incomplete. These factors likely explain the serious inconsistency rating for LOS in the GRADE assessment.

The 37% reduction in postoperative complications associated with ERAS is particularly relevant for older patients, who are at higher baseline risk for both surgical and medical adverse events. The pooled complication endpoint was broad and generally reflected overall postoperative morbidity, including infectious, cardiopulmonary, thromboembolic, urinary, gastrointestinal, and delirium-related events when reported. Interestingly, the subgroup analysis revealed a significantly greater effect in RCTs (RR = 0.52, 48% reduction) compared with prospective cohort studies (RR = 0.67, 33% reduction), with a statistically significant test for subgroup differences (*P* = 0.038). This difference may be attributable to stricter protocol adherence in randomized trials, more rigorous outcome assessment, or residual confounding in observational studies. Nevertheless, both study designs consistently favored ERAS over conventional care.

Several mechanisms may explain the complication-reducing benefits of ERAS. Early mobilization reduces venous stasis and pulmonary complications (41). Multimodal analgesia with opioid-sparing approaches decreases the risk of postoperative delirium, a complication that disproportionately affects older adults (42). The Jia et al. study specifically reported a 55% reduction in postoperative delirium with ERAS (3.4% vs. 12.9%), highlighting this important geriatric-specific benefit (26).

A key concern regarding ERAS implementation in elderly patients has been the potential for increased readmission rates due to premature discharge. Our analysis suggests that ERAS does not increase 30-day readmission rates compared with conventional care (RR = 0.78, *P* = 0.135). This finding is reassuring, but it should be interpreted in the context of variable pathway details, limited compliance reporting, and the fact that discharge thresholds can differ between healthcare systems (43, 44).

The EAES/SAGES 2024 guidelines recommend ERAS implementation for elderly patients undergoing elective colorectal surgery, based on moderate-certainty evidence (14). Our meta-analysis supports this recommendation and extends the evidence base to include hepatobiliary and gastric surgery. However, it is important to note that ERAS protocols may require age-specific modifications. Studies have suggested that elderly patients may benefit from extended preoperative optimization, including prehabilitation programs targeting cardiopulmonary fitness and nutritional status (45, 46).

This study has several strengths. We conducted a comprehensive search across multiple databases and included both RCTs and prospective cohort studies, which broadens external validity and reflects real-world implementation. We also extracted detailed information on ERAS elements, compliance, baseline characteristics, and complication definitions, which is presented in the Supplementary Materials to improve interpretability. However, several limitations should be acknowledged. First, combining randomized and prospective cohort studies may introduce residual bias despite subgroup analyses showing directionally consistent results. Second, heterogeneity in age definitions, surgical procedures, discharge criteria, and ERAS pathway composition limits direct comparability across studies. Third, compliance with ERAS was incompletely reported, preventing a reliable dose-response analysis between pathway adherence and outcomes. Fourth, the complication endpoint was not uniformly defined across studies, although most studies considered overall postoperative morbidity and several used Clavien-Dindo grading. Fifth, we included only English-language publications, which may have introduced language bias. Finally, chronological age was used as the primary eligibility framework, whereas frailty may be more clinically informative than age alone; evidence in patients with marked frailty or age >80 years remains limited (14). Although one hepatectomy trial was not elderly-specific, sensitivity analysis excluding this study did not materially change the pooled estimates (Supplementary Table S5).

Future research should focus on developing and validating age-adapted ERAS protocols, incorporating frailty assessment, standardized reporting of protocol compliance, and procedure-specific analyses for colorectal, hepatobiliary, and gastric surgery. Studies examining long-term functional recovery and quality of life would also strengthen clinical decision-making in older adults.

## Conclusions

This systematic review and meta-analysis suggests that ERAS protocols are effective and generally safe in older patients undergoing major abdominal surgery. ERAS implementation was associated with an approximately 3.3-day reduction in hospital stay and a 37% reduction in postoperative complications, without an increase in 30-day readmission. These benefits were directionally consistent across randomized and prospective cohort studies, but conclusions should be interpreted alongside heterogeneity in procedure type, ERAS implementation, and the limited availability of frailty-specific data.

## Data Availability

The original contributions presented in the study are included in the article/Supplementary Material, further inquiries can be directed to the corresponding author.
