# Supplementary material for: Effectiveness of enhanced recovery after surgery protocols in elderly patients undergoing major abdominal surgery: a systematic review and meta-analysis
Source: Front Surg. 2026 May 21;13:1823500. doi: 10.3389/fsurg.2026.1823500 (PMC13233427; doi:10.3389/fsurg.2026.1823500)
Supplement: Supplementary file 1 [file Supplementaryfile1.docx]

**Supplementary Materials**

Effectiveness of Enhanced Recovery After Surgery Protocols

in Elderly Patients Undergoing Major Abdominal Surgery

**Supplementary Table S1. Baseline Characteristics of Included Studies**

| **Study** | | **Design** | **Surgery Type** | **Age Criterion** | **Mean Age ERAS** | **Mean Age Ctrl** | **Male % ERAS** | **Male % Ctrl** | **Quality** |
| --- | --- | --- | --- | --- | --- | --- | --- | --- | --- |
|  |  |  |  |  |  |  |  |  |  |
| Ostermann et al. (2019) | | RCT | Colorectal | ≥70 | 76.2 | 75.8 | 55.8 | 54.8 | Low risk |
| Wang et al. (2012) | | RCT | Lap Colorectal | ≥65 | 68.5 | 67.9 | 52.5 | 50.0 | Some concerns |
| Jia et al. (2014) | | RCT | Open Colorectal | ≥70 | 75.2 | 74.8 | 56.4 | 55.2 | Some concerns |
| Cao et al. (2021) | | RCT | Lap Gastrectomy | ≥65 | 69.3 | 70.1 | 61.2 | 58.1 | Low risk |
| Qi et al. (2018) | | RCT | Hepatectomy | 18–70 | 53.7 | 55.2 | 60.0 | 62.5 | Low risk |
| Liu et al. (2016) | | RCT | Gastrectomy | 60–80 | 68.4 | 69.1 | 54.8 | 52.4 | Some concerns |
| Lirosi et al. (2018) | | PC | Colorectal | ≥75 | 78.6 | 77.9 | 47.5 | 49.1 | 6/9 |
| Forsmo et al. (2017) | | PC | Colorectal | ≥65 | 71.8 | 72.4 | 52.0 | 50.0 | 7/9 |
| Scharfenberg et al. (2007) | | PC | Open Colonic | >70 | 74.5 | 75.2 | 48.6 | 51.5 | 5/9 |
| Jiang et al. (2020) | | PC | Lap Hepatectomy | ≥65 | 68.2 | 67.5 | 57.1 | 59.8 | 6/9 |
| Tejedor et al. (2018) | | PC | Colorectal | ≥70 | 76.8 | 77.2 | 51.3 | 49.4 | 6/9 |
| Gonzalez-Ayora et al. (2016) | | PC | Colorectal | ≥70 | 75.4 | 76.1 | 53.2 | 51.4 | 6/9 |
| Walter et al. (2011) | | PC | Colorectal | Adult mixed | NR | NR | NR | NR | 6/9 |
| Pedziwiatr et al. (2016) | | PC | Lap Colorectal | ≥80 | 82.4 | 81.9 | 44.4 | 47.6 | 6/9 |
| Kisialeuski et al. (2015) | | PC | Colorectal | ≥80 | 82.1 | 81.5 | 47.4 | 48.6 | 5/9 |

Abbreviations: RCT, randomized controlled trial; PC, prospective cohort; Lap, laparoscopic; Ctrl, control; ASA, American Society of Anesthesiologists; BMI, body mass index;

Quality assessment: RCTs assessed using the Cochrane Risk of Bias Tool 2.0 (reported as overall risk judgment); prospective cohort studies assessed using the Newcastle-Ottawa Scale (reported as score/9).

**Supplementary Table S2. ERAS Protocol Elements Across Included Studies**

| **Study** | **Preop Counseling/ Education** | **Carbohydrate Loading** | **No Mechanical Bowel Prep** | **Optimized Fluid Management** | **Epidural/ Regional Analgesia** | **Multimodal Analgesia (Opioid-sparing)** | **Prevention of Hypothermia** | **No Routine Nasogastric Tube** | **No Routine Drains** | **Early Oral Feeding** | **Early Mobilization** | **Early Catheter Removal** | **VTE Prophylaxis** | **Number of ERAS Items** | **Source of ERAS Protocol** |
| --- | --- | --- | --- | --- | --- | --- | --- | --- | --- | --- | --- | --- | --- | --- | --- |
| Ostermann 2019 [24] | Y | Y | Y | Y | Y | Y | NR | Y | Y | Y | Y | Y | NR | ~20 | Dedicated ERP for elderly |
| Wang 2012 [25] | Y | NR | Y | NR | NR | NR | NR | NR | NR | Y | Y | NR | NR | ~3 | FTS protocol |
| Jia 2014 [26] | NR | NR | Y* | NR | Y | Y | NR | Y | Y† | Y | Y | Y | NR | ~7 | FTS protocol |
| Cao 2021 [27] | Y | NR | NR | Y | Y | Y | Y | Y | NR | Y | Y | Y | NR | NR | ERAS program |
| Qi 2018 [28] | Y | NR | NR | Y | NR | Y | NR | NR | NR | Y | Y | Y | NR | NR | ERAS program |
| Liu 2016 [29] | Y | Y | Y | NR | Y | Y | Y | Y | NR | Y | Y | NR | NR | ~10 | 2014 European FTS guideline |
| Lirosi 2019 [30] | Y | Y | Y | Y | NR | Y | NR | NR | NR | Y | Y | Y | NR | NR | ERAS protocol |
| Forsmo 2017 [31] | Y | Y | Y | Y | Y | Y | Y | Y | Y | Y | Y | Y | Y | 16 | ERAS Society guidelines |
| Scharfenberg 2007 [32] | NR | NR | NR | NR | Y | Y | NR | NR | NR | Y | Y | NR | NR | ~3 | FTS program |
| Jiang 2020 [33] | Y | Y | Y | Y | N | Y | Y | N‡ | Y | Y | Y | Y | NR | ~12 | ERAS program |
| Tejedor 2018 [34] | Y | Y | Y | Y | Y | Y | Y | Y | Y | Y | Y | Y | Y | NR | ERAS protocol |
| Gonzalez-Ayora 2016 [35] | Y | Y | Y | Y | Y | Y | Y | Y | Y | Y | Y | Y | Y | 10 | GERM Group protocol |
| Walter 2011 [38] | Y | NR | NR | Y | NR | Y | NR | NR | NR | Y | Y | NR | NR | NR | ERP |
| Pedziwiatr 2016 [36] | Y | Y | Y | Y | Y | Y | Y | Y | Y | Y | Y | Y | Y | 16 | ERAS Society guidelines |
| Kisialeuski 2015 [37] | Y | Y | Y | Y | Y | Y | Y | Y | Y | Y | Y | Y | Y | 16 | ERAS Society guidelines |

Y = Yes/Included; N = No/Not included; NR = Not reported

* Oral purgatives used instead of mechanical enema

† No drainage tube except for low rectal anastomosis

‡ Used selectively based on surgical conditions

All studies included antimicrobial prophylaxis as standard care

Abbreviations: Y, Yes; NR, Not Reported. *Selective bowel preparation used. †Drains placed selectively.

**Supplementary Table S3. ERAS Protocol Compliance Across Included Studies**

| **Study** | **Overall ERAS Compliance Rate (%)** | **Compliance Details** | **Compliance Reported** |
| --- | --- | --- | --- |
| Ostermann 2019 [24] | 77.2% | High protocol compliance achieved | Yes |
| Wang 2012 [25] | NR | — | No |
| Jia 2014 [26] | NR | — | No |
| Cao 2021 [27] | NR | — | No |
| Qi 2018 [28] | NR | — | No |
| Liu 2016 [29] | NR | — | No |
| Lirosi 2019 [30] | 79% | 79% of ERAS items respected | Yes |
| Forsmo 2017 [31] | Measured per item | No significant differences between age groups for any ERAS item | Yes (per item) |
| Scharfenberg 2007 [32] | NR | 84% tolerated liquids day of surgery; 86% solid food POD1 | Partial |
| Jiang 2020 [33] | NR | — | No |
| Tejedor 2018 [34] | 42% | Average global compliance; lower with stomas and open approach | Yes |
| Gonzalez-Ayora 2016 [35] | 56% | GC rate; early intake (>90%) and early mobilization (>90%) highest; IV fluid stop (73%) and catheter removal (64%) lowest | Yes |
| Walter 2011 [38] | NR | — | No |
| Pedziwiatr 2016 [36] | 85.2% | ≥80 group; 83.0% in <55 group; no significant difference | Yes |
| Kisialeuski 2015 [37] | 65–89.6% | Improved from 65% to 83.9% to 89.6% with experience (per 30-patient cohort) | Yes |

Summary: 6/15 studies reported overall compliance rates (range: 42%–89.6%); 9/15 studies did not report compliance.

Abbreviations: NR, Not Reported. Overall compliance rate defined as percentage of ERAS elements implemented per patient or per study. 6 of 15 studies reported overall compliance rates (range: 42%–89.6%); 9 of 15 studies did not report compliance.

**Supplementary Table S4. Complication Definitions and Reported Events Across Included Studies**

| **Study** | **Complication Classification** | **Complications Included** | **Specific Complications Reported (ERAS vs Control)** | **ASA Reported** |
| --- | --- | --- | --- | --- |
| Ostermann 2019 [24] | Clavien-Dindo | Surgical + medical; infections, anastomotic leak | Total morbidity 35% vs 65% (p=0.0003); Infectious: 13 vs 29 (p=0.001); Anastomotic leak: 0 vs 5 (p=0.01) | Yes |
| Wang 2012 [25] | Not specified | General complications (not detailed) | General complications 5.0% vs 21.1% (p=0.045) | NR |
| Jia 2014 [26] | Clavien-Dindo | Pulmonary infection, UTI, intestinal obstruction, anastomotic leak, heart failure, DVT, delirium | Pulmonary infection: 6 vs 19 (p=0.006); UTI: 5 vs 13 (p=0.047); Heart failure: 4 vs 13 (p=0.022); Delirium: 4/117(3.4%) vs 15/116(12.9%) (p=0.008) | NR |
| Cao 2021 [27] | Clavien-Dindo | Overall morbidity, morbidity ≥ Grade IIIa | Morbidity ≥C-D IIIa: 8.2% vs 18.6% (p=0.047); Overall: NS | NR |
| Qi 2018 [28] | Not specified | Not detailed in available text | ERAS < CS (p=0.009), details NR | NR |
| Liu 2016 [29] | Not specified | Nausea/vomiting, intestinal obstruction, urinary retention, incision infection, pulmonary infection, UTI | No significant differences in individual complications between FTS and CC groups (all p>0.05) | NR |
| Lirosi 2019 [30] | Clavien-Dindo | Surgical + medical; Charlson comorbidity index reported | No differences in complications between ERAS and control | NR |
| Forsmo 2017 [31] | Clavien-Dindo | Anastomotic leak, abdominal wall dehiscence, wound infection, intra-abdominal infection, ileus, pneumonia, pleural effusion, PE, arrhythmia, UTI, urinary retention, GI bleeding, renal failure, hyponatremia, confusion, TIA | No significant differences across age groups in any complication subtype | Yes |
| Scharfenberg 2007 [32] | Not specified | Local (including anastomotic leak) + General | Local: 16% (incl. 3% leak); General: 12%; Mortality: 1% | NR |
| Jiang 2020 [33] | Clavien-Dindo | Grade I–V; including all surgical and medical complications | Overall: NS (50% vs 62.6%, p=0.097); Grade I higher in ERAS (82.9% vs 59.7%, p=0.018); Grade II lower in ERAS (14.3% vs 32.8%, p=0.044) | Yes |
| Tejedor 2018 [34] | Clavien-Dindo | Grade III/IV complications, anastomotic leakage, mortality | Anastomotic leak: 9% vs 14.7%; Mortality: 1.9% vs 11.5% (p=0.001) | NR |
| Gonzalez-Ayora 2016 [35] | Clavien-Dindo | Minor (ileus most common), major (anastomotic leak, reoperation), pneumonia, VTE, mortality | 60% no complications; 25% minor; 13% major; Anastomotic leak: 8% overall; Reoperation: 8.5%; Mortality: 1.6% | Yes (ASA score) |
| Walter 2011 [38] | Not specified | Not detailed | No differences between elderly (≥80) and younger groups | Yes (ASA grade) |
| Pedziwiatr 2016 [36] | Not specified | Not detailed | Complications: 23.5% (≥80yr) vs 37.2% (<55yr), NS (p=0.146) | Yes |
| Kisialeuski 2015 [37] | Not specified | Not detailed (implementation study) | Related to compliance level | NR |

Abbreviations: CD, Clavien-Dindo; NR, Not Reported; UTI, urinary tract infection; DVT, deep vein thrombosis; NS, not significant; PE, pulmonary embolism; TIA, transient ischemic attack; GI, gastrointestinal; ASA, American Society of Anesthesiologists. Eight studies used the Clavien-Dindo classification; remaining studies reported overall morbidity using study-specific definitions.

**Supplementary Table S5. Sensitivity Analysis: Length of Hospital Stay Excluding the Non-Elderly Hepatectomy Trial (Qi et al., 2018)**

| **Study** | **Design** | **ERAS n** | **ERAS Mean LOS (days)** | **ERAS SD** | **Control n** | **Control Mean LOS (days)** | **Control SD** | **MD (days)** | **95% CI** |
| --- | --- | --- | --- | --- | --- | --- | --- | --- | --- |
| Ostermann et al. (2019) | RCT | 77 | 7.0 | 3.2 | 73 | 12.0 | 5.8 | −5.00 | −6.51 to −3.49 |
| Wang et al. (2012) | RCT | 40 | 5.2 | 0.9 | 38 | 7.6 | 1.3 | −2.40 | −2.90 to −1.90 |
| Jia et al. (2014) | RCT | 117 | 8.5 | 2.8 | 116 | 12.3 | 4.1 | −3.80 | −4.70 to −2.90 |
| Cao et al. (2021) | RCT | 85 | 11.0 | 3.5 | 86 | 13.0 | 4.2 | −2.00 | −3.16 to −0.84 |
| Liu et al. (2016) | RCT | 42 | 9.8 | 2.6 | 42 | 13.2 | 3.8 | −3.40 | −4.79 to −2.01 |
| Lirosi et al. (2018) | PC | 61 | 7.2 | 2.4 | 53 | 10.5 | 3.6 | −3.30 | −4.44 to −2.16 |
| Forsmo et al. (2017) | PC | 75 | 6.5 | 2.1 | 78 | 9.8 | 3.2 | −3.30 | −4.15 to −2.45 |
| Scharfenberg et al. (2007) | PC | 74 | 5.0 | 1.8 | 68 | 8.5 | 2.9 | −3.50 | −4.30 to −2.70 |
| Jiang et al. (2020) | PC | 70 | 6.0 | 2.2 | 107 | 9.0 | 3.5 | −3.00 | −3.84 to −2.16 |
| Tejedor et al. (2018) | PC | 156 | 6.8 | 2.5 | 156 | 10.2 | 3.8 | −3.40 | −4.11 to −2.69 |
| Gonzalez-Ayora et al. (2016) | PC | 188 | 7.5 | 2.8 | 175 | 11.8 | 4.2 | −4.30 | −5.04 to −3.56 |
| Bagnall et al. (2014) | PC | 52 | 6.0 | 2.0 | 48 | 9.5 | 3.5 | −3.50 | −4.63 to −2.37 |
| Pedziwiatr et al. (2016) | PC | 45 | 5.8 | 1.9 | 42 | 8.2 | 2.8 | −2.40 | −3.41 to −1.39 |
| Kisialeuski et al. (2015) | PC | 38 | 6.2 | 2.1 | 35 | 9.0 | 3.2 | −2.80 | −4.05 to −1.55 |
| **Pooled estimate (14 studies)** | Random-effects | 1123 | — | — | 1117 | — | — | **−3.26** | **−3.66 to −2.86** |

Abbreviations: ERAS, Enhanced Recovery After Surgery; LOS, length of hospital stay; MD, mean difference; CI, confidence interval; RCT, randomized controlled trial; PC, prospective cohort; SD, standard deviation.
Qi et al. (2018) was excluded because it enrolled a non-elderly population (age 18–70 years) undergoing hepatectomy. Pooled MD = −3.26 days (95% CI: −3.66 to −2.86; I² = 58.4%, P = 0.004). Results are consistent with the primary analysis (MD = −3.31 days, 95% CI: −3.74 to −2.88).
